# Stb6 mediates stomatal immunity, photosynthetic functionality, and the antioxidant system during the *Zymoseptoria tritici*-wheat interaction

**DOI:** 10.3389/fpls.2022.1004691

**Published:** 2022-10-26

**Authors:** Fateme Ghiasi Noei, Mojtaba Imami, Fardad Didaran, Mohammad Amin Ghanbari, Elham Zamani, Amin Ebrahimi, Sasan Aliniaeifard, Mohsen Farzaneh, Mohammad Javan-Nikkhah, Angela Feechan, Amir Mirzadi Gohari

**Affiliations:** ^1^ Department of Plant Pathology, Faculty of Agricultural Sciences and Engineering, College of Agriculture and Natural Resources, University of Tehran, Karaj, Iran; ^2^ Photosynthesis Laboratory, Department of Horticulture, Aburaihan Campus, University of Tehran, Tehran, Iran; ^3^ Department of Horticultural Science, School of Agriculture, Shiraz University, Shiraz, Iran; ^4^ Agronomy and Plant Breeding Department, Faculty of Agriculture, Shahrood University of Technology, Semnan, Iran; ^5^ Department of Agriculture, Medicinal Plants and Drugs Research Institute, Shahid Beheshti University, Tehran, Iran; ^6^ School of Agriculture and Food Science, University College Dublin, Dublin, Ireland

**Keywords:** *Zymoseptoria tritici*, Septoria tritici blotch, resistance gene, stomata immunity, photosynthetic machinery, antioxidant enzymes

## Abstract

This study offers new perspectives on the biochemical and physiological changes that occur in wheat following a gene-for-gene interaction with the fungal pathogen *Zymoseptoria tritici*. The *Z. tritici* isolate IPO323, carries *AvrStb6*, while *ΔAvrStb6#33*, lacks *AvrStb6*. The wheat cultivar (cv.) Shafir, bears the corresponding resistance gene *Stb6*. Inoculation of cv. Shafir with these isolates results in two contrasted phenotypes, offering a unique opportunity to study the immune response caused by the recognition of AvrStb6 by Stb6. We employed a variety of methodologies to dissect the physiological and biochemical events altered in cv. Shafir, as a result of the AvrStb6-Stb6 interaction. Comparative analysis of stomatal conductance demonstrated that AvrStb6-Stb6 mediates transient stomatal closures to restrict the penetration of *Zymoseptoria* *tritici*. Tracking photosynthetic functionality through chlorophyll fluorescence imaging analysis demonstrated that AvrStb6-Stb6 retains the functionality of photosynthesis apparatus by promoting Non-Photochemical Quenching (NPQ). Furthermore, the PlantCV image analysis tool was used to compare the H_2_O_2_ accumulation and incidence of cell death (2, 4, 8, 12, 16, and 21 dpi), over *Z. tritici* infection. Finally, our research shows that the AvrStb6-Stb6 interaction coordinates the expression and activity of antioxidant enzymes, both enzymatic and non-enzymatic, to counteract oxidative stress. In conclusion, the Stb6-AvrStb6 interaction in the *Z. tritici*-wheat pathosystem triggers transient stomatal closure and maintains photosynthesis while regulating oxidative stress.

## Introduction

*Zymoseptoria tritici* (previously known as *Mycosphaerella graminicola*) which causes septoria tritici blotch (STB) is a notorious fungal pathogen limiting wheat production worldwide and threatening global food security (Quaedvlieg et al., 2011). Under favorable conditions, this destructive pathogen leads to significant yield losses and lowers the grain quality (Eyal, 1999). *Z. tritici* is a typical hemibiotroph whose lifestyle constitutes two distinct stages. The asymptomatic biotrophic growth is initiated by germination of the landed conidia on the leaf surface and penetration directly through the stomata. This fungus then colonizes the mesophyll tissue intercellularly without causing visible damage to the wheat host cells. The biotrophic stage lasts 7–10 days, depending on the fungal isolates and wheat genotypes used. This stage is characterized by the induction of weak plant defence (Rudd et al., 2015). Following this stage, *Z. tritici* undergoes the necrotrophic phase, when it begins to release a plethora of cell wall-degrading enzymes to damage plant tissues, eventually leading to the emergence of STB disease. This rapid colonization plays a pivotal role in the build-up of fungal biomass and developing asexual fruiting bodies (pycnidia) (Kema et al., 1996). At the transition phase, energy is redirected away from other photosynthetic areas in the damaged tissue while genes implicated in the photosynthesis process are noticeably down-regulated during the necrotrophic stage (Rudd et al., 2015).

The effector AvrStb6 and wheat resistance protein Stb6 follow the gene-for-gene paradigm for the *Z. tritici*-wheat interaction. The *Z. tritici* IPO323 isolate has the avirulence protein AvrStb6, which is likely recognized by the matching resistance protein Stb6, culminating in host immunity. The IPO323 isolate fails to infect the wheat cv. Shafir harboring *Stb6*, however the IPO323 mutant lacking *AvrStb6* (*ΔAvrStb6#33*) was pathogenic on this cultivar (Kema et al., 2018).

Photosynthesis is a crucial process in plant physiology in which chlorophyll molecules in the chloroplast receive light energy and utilize it to make oxygen and energy-rich chemicals. The biosynthesis of secondary metabolites and defence-related phytohormones such as jasmonic acid and salicylic acid, as well as the regulation of this process, play a critical role in supplying defensive responses to biotic and abiotic stimuli (Kretschmer et al., 2019). Chlorophyll *a* fluorescence (ChlF) is a sensitive and non-invasive tool to investigate the photochemical efficiency of leaves under biotic and abiotic stress. ChlF offers valuable quantitative data for evaluating the photosynthetic performance (Kalaji et al., 2017). This method has been frequently used to study photosynthetic functionality under abiotic (e.g. heavy metal toxicity, drought and salinity stresses) and biotic (fungal attack) stresses (Paunov et al., 2018; Mihailova et al., 2019).

Under cellular stress, chloroplasts overproduce reactive oxygen species (ROS) to counteract adverse circumstances, such as fungal invasion. It’s worth noting that ROS molecules such as H_2_O_2_ have a dual function: they may either operate as signaling molecules that cause programmed cell death or they can stop fungal development by having a direct antifungal effect (Li and Kim, 2021). Under pathogen attack, the attempted infection sites experience an oxidative burst event, characterized by a quick and extensive accumulation of these harmful free radicals. This biological mechanism plays a key role in defining compatibility/incompatibility (Torres, 2010). A previous study demonstrated that ROS molecules accumulated to higher levels in incompatible interactions than in compatible contexts possibly to halt hyphal growth at the biotrophic phase in the* Z. tritici*-wheat interaction. ROS are produced extensively in the compatible interaction to aid the infection process, which coincides with tissue collapse and the production of pycnidia (necrotrophic stage) (Shetty et al., 2003). However, ROS are continually produced at a low level in chloroplasts during photosynthesis, which is unharmful to live cells because multiple antioxidant enzymes maintain a balance between synthesizing and detoxifying ROS molecules (Apel and Hirt, 2004).

This study compares the physiological and biochemical changes in cv. Shafir inoculated by *Z. tritici* IPO323 and the knockout mutant lacking *AvrStb6* (*ΔAvrStb6#33*). (Kema et al., 2018). The ChlF technique and microscopic analysis were used to evaluate the impact of the AvrStb6-Stb6 interaction on the photosynthetic functionality and stomatal-related features, respectively. We also employed DAB and trypan blue staining techniques in conjunction with the image analysis program PlantCV to identify and quantify the amount of H_2_O_2_ accumulated in infected tissues and/or the occurrence of cell death. Oxidative stress was measured by changes in levels of malondialdehyde (MDA) and electrolyte leakage (EC). Five phenolic compounds were investigated as non-enzymatic antioxidant agents using high-performance liquid chromatography (HPLC) in both examined interactions. Proline which has antioxidant activity, was evaluated (Hayat et al., 2012). The activities of five enzymatic antioxidant agents, including the superoxide dismutase (SOD), Catalase (CAT), Guaiacol peroxidase (GPX), Ascorbate peroxidase (APX), and Glutathione reductase (GR) were also investigated during infection with IPO323 and *ΔAvrStb6#33.*

Measurements were undertaken (2, 4, 8, 12, 16 and 21 dpi) corresponding to three phases of *Z. tritici* infection; the biotrophic stage (2-4 dpi)), the transition from biotrophy to necrotrophy (8 dpi), and the necrotrophic stage (12, 16, and 21 dpi). This study revealed that *Stb6* in the cv. Shafir triggers transient stomatal closure and maintains photosynthesis while regulating oxidative stress.

## Material and methods

### Biological materials

Throughout the experiments, *Z. tritici* IPO323 (WT) and the corresponding mutant strain *ΔAvrStb6#33* were utilized (Kema et al., 2018). Both strains were stored at -80°C before being re-cultured at 18°C on a V8 juice medium (Campbell Foods, Puurs, Belgium). Yeast Malt Dextrose Broth (YMDB) medium (Yeast extract 4 g/L, Malt 4 g/L, and Dextrose 4 g/L) was used to abundantly generate yeast-like spores by growing the strains on this medium and placing the inoculated flasks in an orbital shaker (Innova 4430; New Brunswick Scientific, Nijmegen, The Netherlands) at 18°C for 5-7 days. The wheat cultivar Shafir, which carries the resistance gene *Stb6*, was employed in the infection assay (Mirzadi Gohari et al., 2014; Saintenac et al., 2018). Wheat cv. Shafir infected with IPO323 were designated as incompatible interaction, while those inoculated with *ΔAvrStb6#33* were marked as a compatible interaction.

### Stomatal measurement

Stomatal morphological features (including stomatal length, stomatal width, pore aperture, stomatal index, and density), were first calculated using the previously described protocol (Aliniaeifard and Van Meeteren, 2016). Subsequently, stomatal conductance (g_s_) of the inoculated and non-inoculated leaves surfaces were measured at 2,4, 8, 12, 16, and 21 dpi by following the protocol described previously (Fanourakis et al., 2013). Based on the assumption that guard cells inflate to form a circular cross-section, the stomatal pore depth was calculated as being equal to the guard cell width (stomatal width/2). On three randomly chosen areas of the same leaf, stomata were counted and their sizes measured. A total of twelve leaves derived from four biological samples were examined and this was repeated independently twice. One square millimeter of leaf tissue was analyzed on both sides of the main vein. The following equation was utilized to calculate g_s_ (Fanourakis et al., 2013):


$$\left[gs=\;\frac{\left(diffusion\;coefficient)\times (stomatal\;density)\times (\pi \;\times poreapperture÷2\;\times \;porelength\;÷2\right)}{(molar\;volume\;of\;air)\;\times [(pore\;depth)+\;\sqrt{\left(pore\;apperture\;÷2\;\times pore\;length\;÷2\right)}]\;}\right]$$

### Polyphasic chlorophyll fluorescence transients

The OJIP transients of dark-adapted (20 min) plants were measured using a Fluorpen FP 100-MAX (Photon Systems Instruments, Drasov, Czech Republic) at 2, 4, 8, 12, 16, and 21 dpi. The OJIP protocol was used to investigate biophysical and phenomenological parameters related to plant stress and photosystem II (PSII) status (listed in **Supplementary Table 1**), as described previously (Strasser et al., 2000). The energy fluxes of light absorption (ABS) and trapping (TR) of the excitation energy, as well as electron transport (ET_O_) per reaction center (RC), are described in **Supplementary Table 1** using parameters derived from the OJIP protocol.

### Chlorophyll fluorescence imaging analysis

A FluorCam device (FluorCam FC 1000-H, Photon Systems Instruments, PSI, Czech Republic) was employed to image the chlorophyll fluorescence of dark-adapted (20 min) plants at multiple time points, including 2, 4, 8, 12, 16, and 21 dpi. Maximum quantum yield of photosystem II (F_V_/F_M_) was determined using a custom protocol depicted previously (Shomali et al., 2021). Measurements of chlorophyll fluorescence began with the samples being exposed to short flashes in darkness, followed by a saturating pulse for longer duration of 3900 mol m^-2^ s^-1^ Photosynthetic photon flux density (PPFD) at the end of the measurement to stop electron transport due to Quinone acceptor reduction (Genty et al., 1989). Two sets of fluorescence data were recorded using the applied protocol: one averaged over the time of short flashes in the dark (Fo) and the other at the time of exposure to saturating flash (F_M_). Maximum fluorescence in light-adapted steady-state (F_M’_) was measured to determine the non-photochemical quenching (NPQ). FluorCam software version 7 (PSI, Czech Republic) was used to analyze the data. The Fv/F_M_ was estimated through the following equation: Fv/F_M_= (F_M_-F_O_)/F_M._ Additionally, the NPQ was calculated based on the following equation: NPQ = (F_M_/F_M_’)-1.

### *In planta* detection of H_2_O_2_ and cell death

Detection of H_2_O_2_ *in planta* was conducted through staining by 3,3 diaminobenzidine (DAB, D-8001, Sigma) (Thordal‐Christensen et al., 1997). The inoculated leaves were harvested at 2, 4, 8, 12, 16, and 21 dpi, and placed in a container filled with acidic water (pH 3.8), containing 1 mg/ml DAB under darkness. The container was kept in a desiccator equipped with a vacuum pump to generate a suction force of 0.2 bar for 30 min, and this was maintained overnight. Following the next day, a clearing procedure to eliminate chlorophyll was conducted using absolute ethanol/acetic acid/glycerol (3:1:1) for 30 min at 80 °C (Shetty et al., 2003). Dead cells and fungal structures were stained using trypan blue based on the procedure reported by Koch and Slusarenko (1990) (Koch and Slusarenko, 1990). These assays were repeated independently three times.

### Image processing

The integrated optical density (IOD) of leaves areas stained either by DAB or trypan blue was obtained using PlantCV (Gehan et al., 2017). The multiclass naive Bayes approach based on RGB pixel values was utilized for this purpose. The classes included background, unstained, and three staining intensities (low, medium, and high). These classifications are based on how intensely the colour is present in the stained tissues; these intensities were first visually classified, and then the colour spectrum was detected using Photoshop software. For machine learning to define the three intensities level, a table of red, green, and blue color values was created from 50 pixels in each class, with each column of the table allocated to a specific class (Díaz‐Tielas et al., 2012). Ultimately, thousands of pixels in all leaves were sorted into these classes using machine learning data. The table was used to generate probability density functions (PDFs) for each of the classes. The RGB values were extracted from the leaf images using Adobe Photoshop (Version 23.1.0).

### Infection biology

Inoculated leaves of the wheat cv. Shafir was collected at 2, 4, 8, 12, 16, and 21 dpi to investigate the infection biology of *Z. tritici* in both compatible and incompatible interactions. At each sampling time, a total of twelve leaves derived from four biological samples were harvested, followed by clearing using absolute ethanol/acetic acid/glycerol (3:1:1) for 30 min at 80 °C to eliminate the natural pigment of the examined leaves (Shetty et al., 2003). Four biological samples were used in this assay, which was independently performed twice. Afterward, microscopic slides were prepared from the inoculated part of the leaves using lactophenol solution. The prepared slides were examined through an Olympus^®^ light microscope (Olympus, Tokyo, Japan). Studying the quantitative developmental stages of employed strains *in planta* was conducted based on the previously reported protocol (Shetty et al., 2003).

### Antioxidant enzymes activity

Harvested samples were homogenized in an extraction buffer comprising 15% acetic acid and 85% methanol. These homogenates were centrifuged at 12,000 rpm for 15 minutes at 4°C. The resulting supernatant were filtered with a 0.45 μm disposable syringe, and this solution was used to measure antioxidant enzyme activity. The superoxide dismutase (SOD) activity was determined based on its inhibition in the photoreduction reduction by the nitroblue tetrazolium (NBT). The final reaction was read spectrophotometrically through a spectrophotometer device (UV-1800; Shimadzu Corporation, Kyoto, Japan) at a wavelength of 560 nm as documented earlier (Beauchamp and Fridovich, 1971). Catalase (CAT) activity was measured spectrophotometrically, and the absorbance recorded at 240 nm (Ranieri et al., 2003). Guaiacol peroxidase (GPX) activity was estimated at 25°C through a spectrophotometer tool adjusted at 470 nm (Hemeda and Klein, 1990). Ascorbate peroxidase (APX) activity was measured by following the procedure depicted previously (Ranieri et al., 2003). Finally, the Glutathione reductase (GR) activity was spectrophotometrically estimated at 412 nm (Smith et al., 1988).

### HPLC-UV analysis of phenolic compounds

As previously reported, the HPLC profile analysis of methanolic extracts was performed using a Waters 2695 Alliance HPLC system with a 996 PDA detector to monitor phenolic compounds (Mansoor et al., 2020). The gradient programme was run for 60 min using (A) methanol+ 0.02 percent TFA; and (B) HPLC grade water+ 0.02 percent TFA, with a flow rate of 0.5 mL min^-1^ on the C18 column (Novapack C18, 4.6 15 mm, 4 m). Peaks were monitored at wavelengths of 200-400 nm, and each phenolic compound was identified by comparing its spectra and retention time to standards. The external standard method was used to make quantitative determinations with commercial standards. Results are expressed as parts per million (ppm). Here, we traced five phenolic compounds in the investigated interactions as described in **Supplementary Table 2**.

### Proline content

The reported procedure was applied to measure the proline content (Bates et al., 1973). Briefly, 300 mg of leaves were homogenized in 10 ml of 3% sulphosalicylic acid. The solutions were centrifuged at 2,000 g for 5 min. The extract was then mixed with acidic-ninhydrine and glacial acetic acid for 45 min at 100°C. Toluene was used to extract the reaction mixture. Separated toluene’s chromophore. Using a UV–Vis spectrophotometer, 515 nm absorbance was read (UV-1800; Shimadzu Corporation, Kyoto, Japan).

### Disruption of membrane integrity

To investigate disruption in cell membrane integrity, malondialdehyde (MDA) content and electrolyte leakage were measured. MDA content was calculated as reported previously with minor modification (Stewart and Bewley, 1980). Harvested samples (0.25 g) were homogenized in 5% (w/v) trichloroacetic acid (TCA) and centrifuged at 10000 rpm for 15 min. Sample supernatant (1 mL) was mixed with 5 mL of trichloroacetic acid containing 0.5% thiobarbituric acid (TBA). The mixture was boiled at 95°C for 30 min. The absorbance at 532 and 600 nm was measured. The non-specific absorbance at 600 nm was subtracted from what was measured at the absorbance of 532 nm. The concentration of MDA was measured *via* the extinction coefficient of 155 mM^−1^ cm^−1^. The electrolyte leakage was estimated by applying the previously documented protocol (Bajji et al., 2002). Briefly, five leaves with 10 ml distilled water were punched. The containers were shaken in an orbital shaker for 24 h at 150 rpm, and the electrolyte conductivity (EC0) was read. The leaves were autoclaved at 120°C for 20 min to determine the maximum leakage, and immediately, electrolyte conductivity (EC1) was estimated after cooling. Eventually, the percentage of leaf electrolytes leakage (EL) was calculated as documented previously (Bajji et al., 2002).

### Statistical analysis

The data were analyzed using SAS software (version 9.0). The two-way analysis of variance (ANOVA) was used to find the significant differences (p ≤ 0.05) and then the Duncan multiple comparisons test was used to compare the means. For analyzing chlorophyll fluorescence parameters, obtained data were subjected to two-way ANOVA, and for mean comparison, the Tukey multiple comparison tests were used. For stomatal characteristics, data was collected from each single leaf on three randomly chosen areas of the same leaf, stomata were counted and their sizes measured. Stomatal features were deemed to be non-independent, and a two-way ANOVA and Tukey multiple comparison tests were used to compare mean values. Odds ratios were calculated for comparison of the variables (percentages) using cv. Shafir inoculated by *ΔAvrStb6#33* as a reference, as previously reported (Shetty et al., 2003). The spider plots were produced using Microsoft Excel 2020, which aided in the accurate evaluation of the parameters gathered from the OJIP data. For all analyses, we assumed a significance level of 0.05.

## Results

### AvrStb6-Stb6 establishes resistance reaction

The cultivar Shafir which carries *Stb6* (Zhong et al., 2017) was inoculated by IPO323 and *ΔAvrStb6#33* strains to validate the impact of *AvrStb6* deletion on STB development. Typical STB symptoms, including the emergence of small chlorotic specks formed specifically at the tip of the leaves, which become visible at 8 dpi in plants challenged by the *ΔAvrStb6#33* strain while no symptoms appeared in the plants inoculated by IPO323. In the compatible interaction, the chlorotic lesions expanded into larger areas at 12 dpi and, subsequently, coalesced into typical necrotic STB blotches bearing numerous pycnidia at 16 dpi (**Supplementary Figure 1A**). At 21 dpi, the inoculated leaves became completely necrotic and covered by abundant asexual fruiting bodies; the necrotic leaf area was 100% ± 0 and pycnidia formation was 89% ± 1 following *ΔAvrStb6#33* infection while no pycnidia were formed on plants inoculated by IPO323 (**Supplementary Figures 1B, C**).

### AvrStb6-Stb6 mediates transient stomatal closures

**Figure 1:**
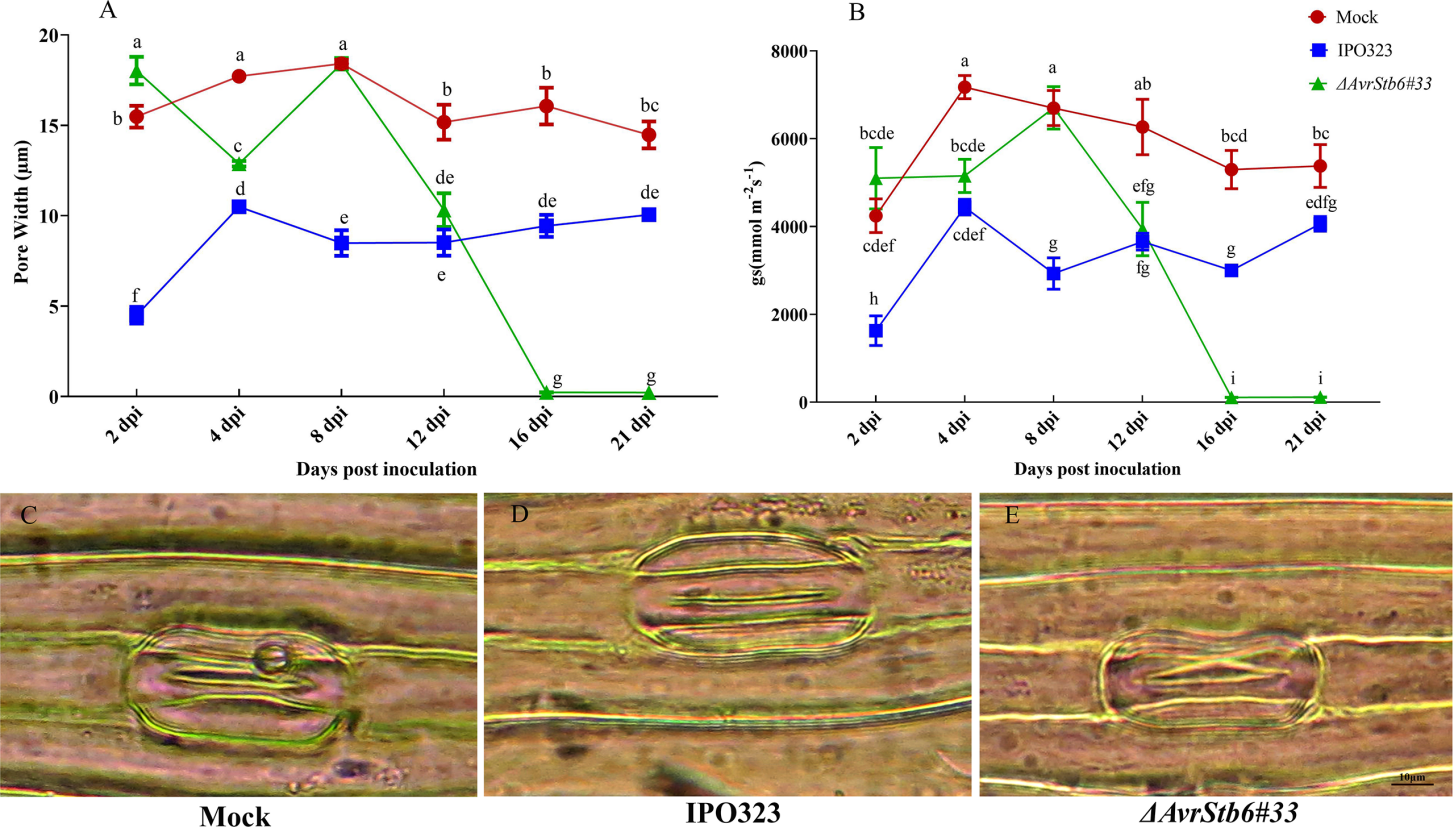
The effect of AvrStb6-Stb6 interaction on stomatal pore width and gas exchange. **(A)** Measuring the stomatal pore width of cv. Shafir inoculated by distilled water (Mock), the WT strain carrying the *AvrStb6* (IPO323), and deleted mutant for *AvrStb6* (*ΔAvrStb6#33*); **(B)** estimating the g_s_ ratio of the treated plants; **(C)** Microscopic picture of a stomata from a plant treated by distilled water (Mock: semi-open); **(D)** inoculated by IPO323 (closed), and **(E)** inoculated by *ΔAvrStb6#33* (open). Data indicate mean ± SD (Standard Deviation) of twelve individual leaves derived from four biological samples. This assay was repeated independently twice. Different lowercase letters indicate statistically significant differences (P≤0.05).

We investigated the stomatal pore width and stomatal conductance (g_s)_ of treated plants at 2, 4, 8, 12, 16, and 21 dpi to explore stomatal function as a defensive response (Grimmer et al., 2012) towards *Z. tritici* infection. At 2 dpi, the highest pore width was observed in plants infected by *ΔAvrStb6#33*, while the lowest was found for IPO323-inoculated plants. Following the transition stage (8 dpi), the pore width of resistant plants remained constant without further fluctuations by 21 dpi, while that of susceptible plants underwent a dramatic reduction to almost zero at later time points (16 and 21 dpi). The g_s_ ratio followed the same pattern as described for the stomatal pore width. For instance, the g_s_ of resistant plants declined by 32% at 2 dpi, whereas that of mock and susceptible plants remained at the same level, which was around 5000 mmol m^−2^ s^−1^. Therefore, our microscopic observation at 2 dpi demonstrated that stomatal closure occurred only in plants infected with IPO323, whereas stomata were semi-open in mock treated and open in plants inoculated by isolate *ΔAvrStb6#33* (**Figure 1**).

### AvrStb6-Stb6 interaction maintains photosynthetic functionality primarily by maintaining NPQ

**Figure 2:**
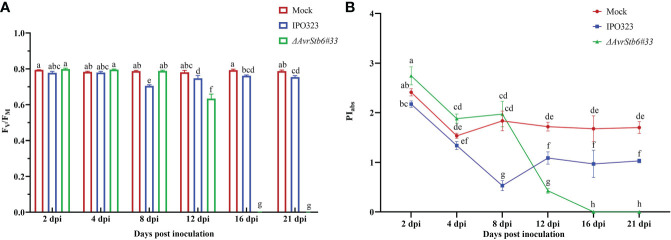
The impact of AvrStb6-Stb6 relationship on the maximum quantum yield of PSII (Fv/F_M_) **(A)** and Performance index (PI_abs_) **(B)** parameters. The leaves of cv. Shafir harboring Stb6 were inoculated with the WT IPO323 and *AvrStb6#33* strains. The essential parameters to calculate the Fv/F_M_ and PI_abs_ were measured by a Fluorpen FP at several time points, including 2, 4, 8, 12, 16, and 21 days post-inoculation. Data are indicate as mean ± SD (Standard Deviation) of four biological samples from one independent experiment. Different lowercase letters indicate statistically significant differences (P≤0.05).

**Figure 3:**
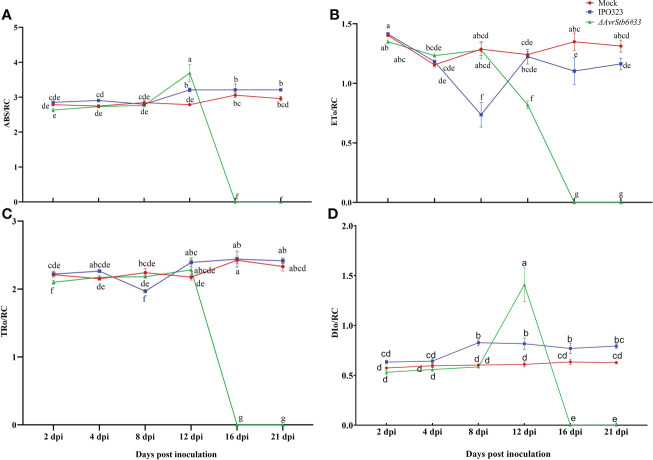
AvrStb6-Stb6 interaction impacts ABS/RC **(A)**, ET_0_/RC (L688) **(B)**, TR_0_/RC (L688) **(C)**, and DI_0_/RC (L688) **(D)** H_2_O_2_ (L897)parameters. The WT IPO323 and *ΔAvrStb6#33* strains were applied to infect the leaves of cv. Shafir harboring the Stb6. The Fluorpen FP instrument was used to measure the key parameters for calculating the ABS/RC **(A)**, ET_0_/RC **(B)**, TR_0_/RC **(C)**, and DI_0_/RC **(D)** parameters at multiple days post-inoculation, including 2, 4, 8, 12, 16, and 21 days. Data are indicate as mean ± SD (Standard Deviation) of four biological samples from one independent experiment. Different lowercase letters indicate statistically significant differences (P≤0.05).

Since *Z. tritici* causes chlorosis and necrotic lesions on wheat leaves, we speculate it may affect the hosts photosynthetic apparatus. The photosynthetic functionality following inoculation with IPO323 and *ΔAvrStb6#33* (incompatible and compatible interactions, respectively) was recorded. F_O_, F_i_, F_j_, F_v_ and F_m_ dropped significantly from 78.8-100% in the cv. Shafir inoculated by *ΔAvrStb6#33* compared to those of plants infected by IPO323 at 16 and 21 dpi (**Supplementary Table 3**). At 8 dpi, the F_V_/F_M_ ratio, as an indicator of PSII’s maximum quantum efficiency, was slightly reduced (9.5% from 4 dpi) in plants infected by IPO323, but this ratio increased at 12 dpi (6% from 8 dpi) and remained constant between 12 to 21 dpi. However, in contrast, the F_V_/F_M_ ratio was drastically reduced in compatible interactions following *ΔAvrStb6#33* infection. As a result, following 12 dpi the ratio (0.62) decreased to zero at 16 and 21 dpi (**Figure 2A**). The PI_abs_ (photosynthetic performance index) was significantly higher at 2dpi in the Mock treated plants and those inoculated with *ΔAvrStb6*#33 compared to IPO323 inoculated plants at 2 dpi. The PI_abs_ significantly decreased in plants inoculated with *ΔAvrStb6*#33 from 8 dpi (1.97) to 0 at 16 dpi. During the incompatible interaction (plants inoculated with IPO323), PI_abs_ decreased 50% at 8 dpi (0.52) compared with that of this index measured at 4 dpi (1.34). Following that, this index recovered at 12 dpi and remained stable until 21 dpi (**Figure 2B**). In the compatible interaction (plants inoculated with *ΔAvrStb6*#33), the highest ABS/RC and DI_0_/RC peaked at 12 dpi before falling to 0 at 16 dpi. While ET_0_/RC and TR_O_/RC parameters suddenly decreased at 16 dpi during the compatible interaction (**Figures 3A-D**). In contrast during the incompatible interaction, plants inoculated with IPO323 had a relatively constant ABS/RC and DI_0_/RC with a small but significant increase between 8 dpi and 12 dpi. The ET_0_/RC gradually declined from 2 dpi to 8 dpi before recovering to a similar level to that found for the compatible interaction at 12 dpi and remaining stable until 21 dpi (**Figure 3B**). TR_O_/RC was significantly reduced in the incompatible interaction at the transition stage (8 dpi), but at 12 dpi returned to levels previously found at 2 and 4 dpi (**Figure 3C**).

The efficiency for electron transfer (Ф_E0_) and energy dissipation (Ф_D0_) were computed to evaluate the energy flow in the photosynthesis process of two studied interactions. The Ф_E0_ was reduced by 36% in the incompatible interaction between 4 and 8 dpi. Afterward, the Ф_E0_ was elevated by 36% between 8 and 12 dpi returning to previous levels and remaining unaltered until 21 dpi. The Ф_E0_ during the compatible interaction dropped by 52% between 8 and 12 dpi, thereafter it had completely stopped by 16 dpi (**Supplementary Table 3**). The highest Ф_D0_ was found in compatible interactions at 16 and 21 dpi, while no fluctuations were found for the mock treated plants throughout (**Supplementary Table 3**). All of the parameters derived from the chlorophyll fluorescence OJIP curves are presented as a spider plot (**Supplementary Figure 2**).

**Figure 4:**
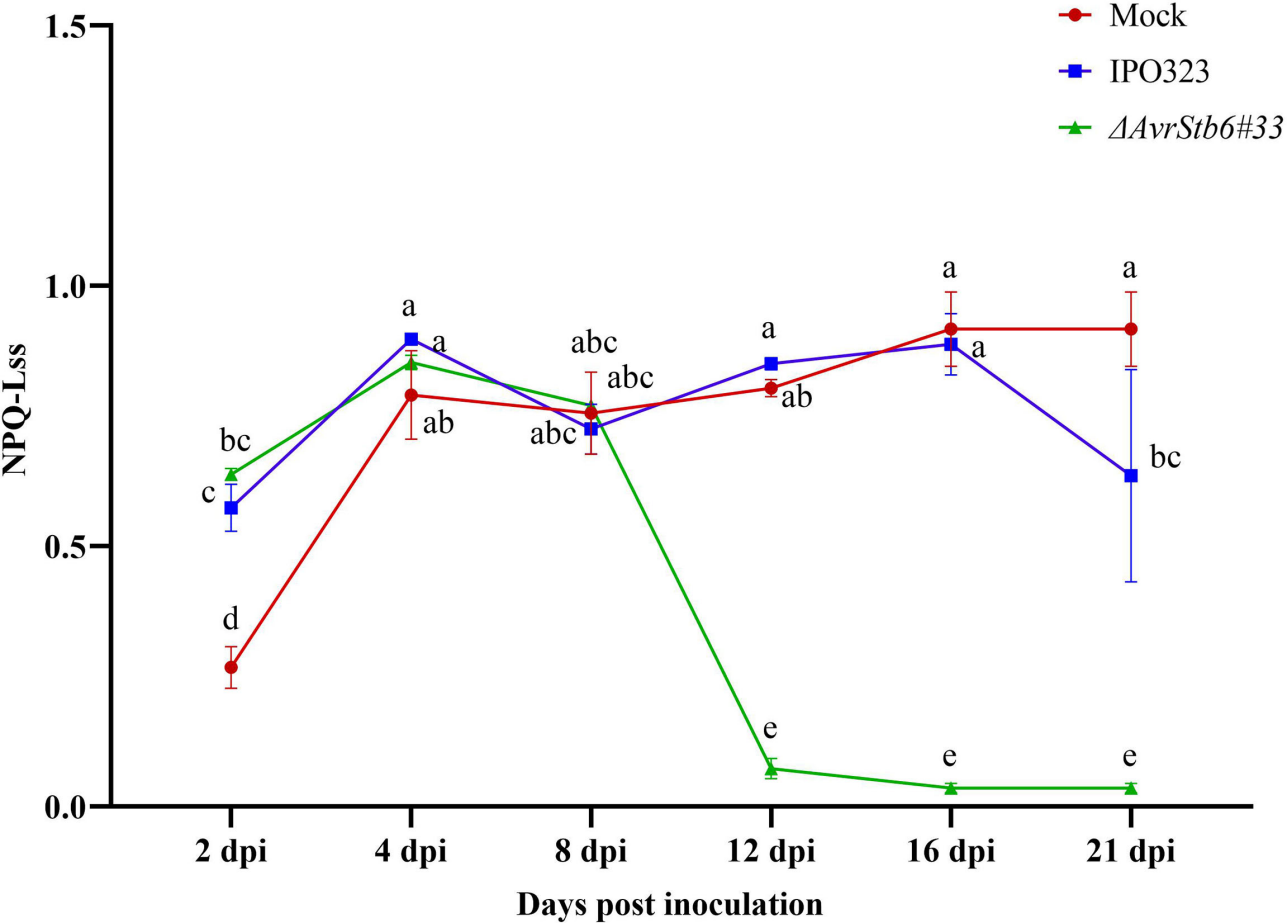
AvrStb6-Stb6 interaction leads to high non-photochemical quenching (NPQ). The leaves of cv. Shafir with Stb6 was infected with either the WT IPO323 and *ΔAvrStb6#33* strains and NPQ was recorded at 2, 4, 8, 12, 16, and 21 days post-inoculation. Data are indicate as mean ± SD (Standard Deviation) of four biological samples from one independent experiment. Different lowercase letters indicate statistically significant differences (P≤0.05).

The release of energy in the form of heat and fluorescence (NPQ) increased steadily in all interactions, from 2-4 dpi. After 8 dpi, there was a sudden and sharp decrease in NPQ (a 95% decrease) in the compatible interaction between 8-12 dpi, remaining low without significant changes until 21 dpi. In the incompatible interaction, the NPQ began to rise slowly, by 26.2% between 2-4 dpi and fell 20% between 4-8 dpi. Following the switching stage at 8 dpi, there was an increase of 17% where this ratio reached similar levels to that of control plants at 12 and 16 dpi. The NPQ fell again by 25% between 16 dpi and 21 dpi (**Figure 4**). Modification occurred in the photosynthetic parameters and chlorophyll fluorescence images of infected wheat leaves taken at 2, 4, 8, 12, 16, and 21 dpi were presented in the **Supplementary Figure 3**.

### AvrStb6-Stb6 regulates H_2_O_2_ accumulation and cell death occurrence

**Figure 5:**
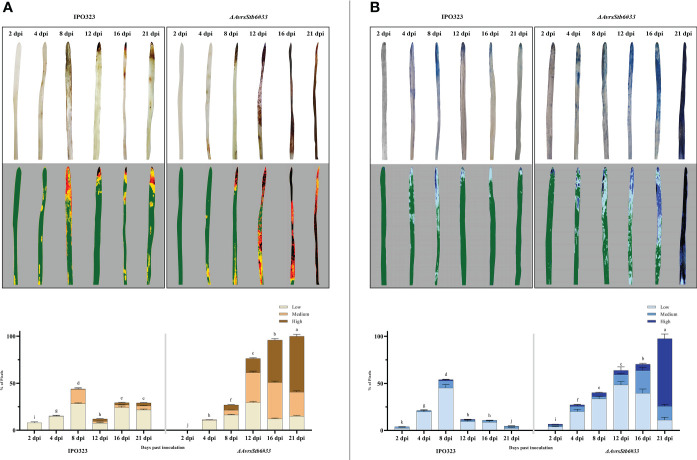
AvrStb6-Stb6 interaction affects both the accumulation of hydrogen peroxide (H_2_O_2_) following staining by DAB signified by the red-brown areas **(A)** and the occurrence of cell death indicated as dark-blue areas stained by the trypan blue **(B)**. Pictures were taken at 2, 4, 8, 12, 16, and 21 dpi. The images were analyzed by the PlantCV software to obtain the integrated optical density (IOD), displaying the percentage of the stained cells by DAB and trypan blue. Data are indicate as mean ± SD (Standard Deviation) of four biological samples from three independent experiments. Different lowercase letters indicate statistically significant differences (P≤0.05).

We histochemically compared the accumulation of H_2_O_2_ and the onset of cell death at several time points, including 2, 4, 8, 12, 16, and 21 dpi, to assess the role of H_2_O_2_ and cell death in two studied interactions. In the incompatible interaction, the timing and accumulation of H_2_O_2_ and cell death was found at low levels 2-4 dpi peaking at 8 dpi (**Figure 5**). Following the switch (8 dpi), no extensive generation and localization of H_2_O_2_ or widespread cell death were observed at the necrotrophic stage (12, 16, and 21 dpi). Plants infected with the *ΔAvrStb6#33* (compatible interaction), on the other hand, showed steady H_2_O_2_ accumulation and cell death from 2 dpi onwards displaying high levels of H_2_O_2_ accumulation and the occurrence of cell death at the necrotrophic growth stage (12, 16, and 21 dpi). Therefore, in the compatible interaction, dramatic and uncontrolled H_2_O_2_ accumulations occurs, leading to the complete death of the inoculated leaves at 21 dpi. The PlantCV program was also used to quantify the stained area of infected leaves by DAB. Our findings demonstrated that the incompatible interaction produces substantially more H_2_O_2_ than the susceptible plants during the transition stage (8 dpi) (**Figure 5A**). Similar results were achieved for trypan blue staining and the PlantCV tool. At 8 dpi, substantial cell death occurred in the incompatible interaction compared with the compatible interaction. Additionally, high levels of cell death occurred following infection of wheat with *ΔAvrStb6#33* at the necrotrophic stage (**Figure 5B**).

### AvrStb6-Stb6 blocks fungal growth and pycnidial formation

**Figure 6:**
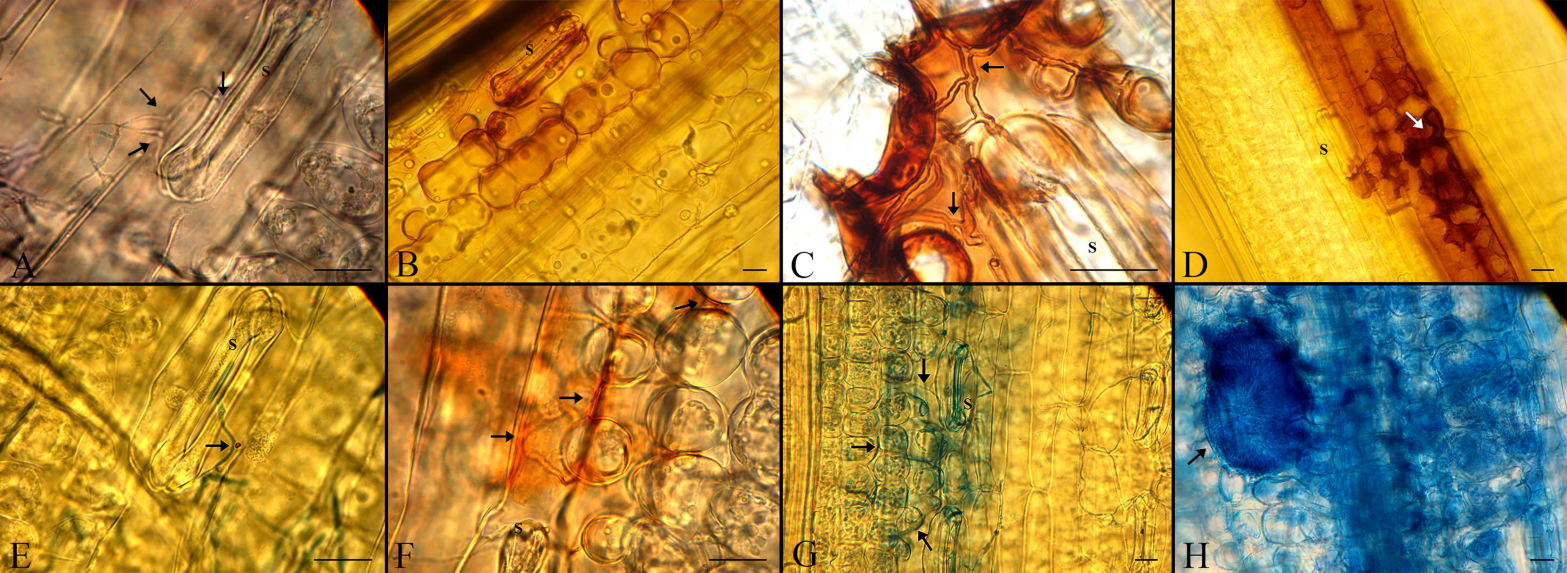
Histopathological events in the cv. Shafir infected by either the WT IPO323 (incompatible interaction) or the ΔAvrStb6#33 (compatible interaction) strains. **(A, E)** Both fungal strains germinated by 2 dpi and penetrated directly through stomata (S stands for the stomata and arrows represent the germinated spores). **(B, F)** Accumulation of H_2_O_2_ as seen by the formation of red-brown staining in the incompatible (upper panel) and compatible interactions (lower panel), respectively at 4 dpi. **(C, G)** Extensive accumulation of H_2_O_2_ in the cv. Shafir infected by IPO323, blocks further hyphal growth while hyphal growth of *ΔAvrStb6#33* was found in the cv. Shafir at 8 dpi. **(D)** Cytoplasmic-like shrinkage occurred in the incompatible context at 8 dpi. **(H)** A-sexual flask-shaped fruiting buddings were formed in the compatible interaction at 12 dpi. The scale bars are 20 μm.

The spores of the employed strains germinated by forming thin germ tubes penetrated directly through stomata by 2 dpi (**Figures 6A–E**). The percentage of spores that germinated was low (15–29%) (**Supplementary Table 4**), but it did not differ significantly between the compatible and incompatible interaction at each of the time-points. The directions of germ tubes derived from germinating spores were divided into three categories, but there were no significant differences in the number of germ tubes in each category between the two interactions, as shown in **Supplementary Table 4**. In the incompatible interaction, H_2_O_2_ accumulated in stained cells 2-8 dpi to a higher level than found in plants inoculated by the *ΔAvrStb6#33* strain (**Figures 5A**, **6B–F**). This occurred particularly around the substomatal cavity where penetration happened (**Figure 6B**). H_2_O_2_ accumulated around the fungal hyphae starting to move into mesophyll cells, and no hyphal growth beyond the H_2_O_2_ accumulation was observed. In the compatible interaction, hyphal growth was found at the switching phase (8 dpi) possibly as the accumulated H_2_O_2_ was inadequate to suppress the hyphal progression (**Figure 6G**). Cell collapse associated with cytoplasmic-like shrinkage was also found in the incompatible context at 8 dpi (**Figure 6D**). Massive H_2_O_2_ accumulation was observed at 12 dpi, coinciding with the pycnidial formation and tissue collapse. Mature asexual fruiting bodies (**Figure 6H**) along with cell death of plant tissues were found at 16 and 21 dpi, respectively.

### AvrStb6-Stb6 manipulates the activities of enzymatic antioxidant agents

**Figure 7:**
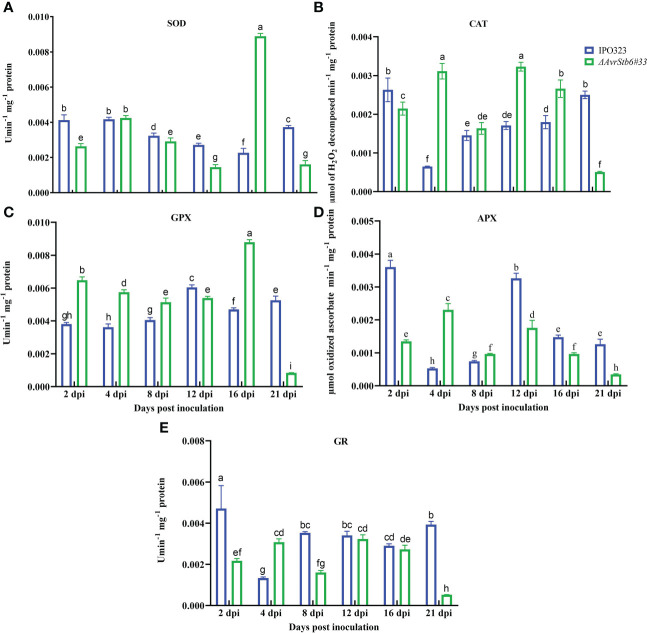
AvrStb6-Stb6 interaction manipulates the activities of five enzymatic antioxidant agent. Activities of five antioxidant enzymes: **(A)** superoxide dismutase (SOD), **(B)** Catalase (CAT), **(C)** Guaiacol peroxidase (GPX), **(D)** Ascorbate peroxidase (APX), and **(E)** Glutathione reductase measured spectrophotometrically following infection of cv. Shafir by WT IPO323 or *ΔAvrStb6#33* strains. Data are indicate as mean ± SD (Standard Deviation) of four biological samples from one independent experiment. Different lowercase letters indicate statistically significant differences (P≤0.05).

Since H_2_O_2_ accumulation was observed in the interaction, the activities of five enzymatic antioxidant agents (e.g. SOD, CAT, GPX, APX, and GR) were measured following infection by the employed strains to examine their potential role in the pathosystem, complying with the *AvrStb6-Stb6* relationship. SOD activity was significantly higher in the incompatible interaction than in the compatible interaction at all-time points, except for 4 and 16 dpi, while SOD was highest in the compatible interaction at 16 dpi (**Figure 7A**). CAT was considerably activated in the compatible interaction as compared to the incompatible interaction except at 4 and 12 dpi (**Figure 7B**). GPX was notably activated at 16 dpi in the compatible interaction compared to the incompatible context where GPX was activated significantly at 21 dpi (**Figure 7C**). The highest APX activity peaks occurred at 2 and 12 dpi in the IPO323-infected plants, whereas lowest peak occurred at 21 dpi in plants infected with *ΔAvrStb6#33* (**Figure 7D**). The GR activity showed high initial and final values (at 2 and 21 dpi) in the incompatible interaction compared with the compatible interaction (**Figure 7E**). At 21 dpi, the activity of all antioxidant enzymes in the compatible interaction was consistently retarded and clearly diminished, which is indicative of an abated antioxidant defence system of the plant.

### AvrStb6-Stb6 orchestrates the activities of non-enzymatic antioxidant agents

**Figure 8:**
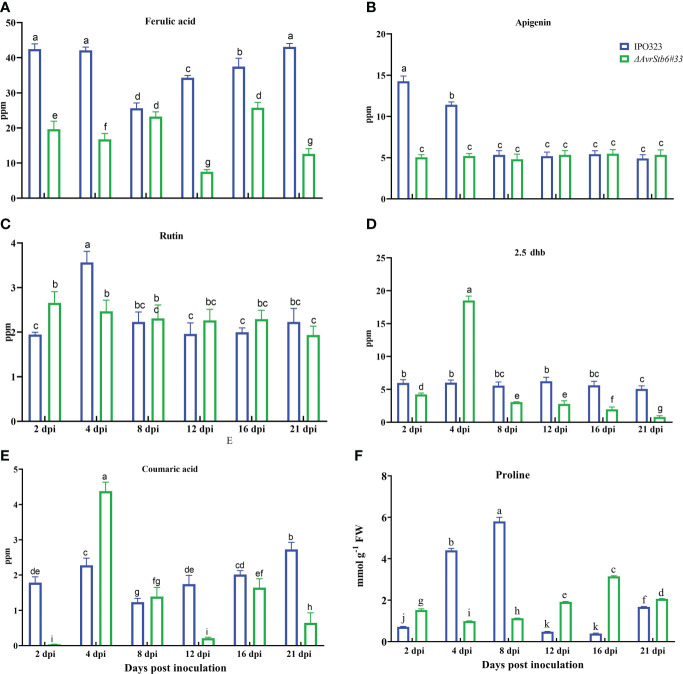
AvrStb6-Stb6 interaction orchestrates the activities of six non-enzymatic antioxidant agent: **(A)** Ferulic acid, **(B)** Apigenin, **(C)** Rutin, **(D)** 2,5-Dihydroxybenzoic aci (2.5 dhb), and **(E)** coumaric acid, and **(F)** Proline. Data are indicate as mean ± SD (Standard Deviation) of four biological samples from one independent experiment. Different lowercase letters indicate statistically significant differences (P≤0.05).

Our findings show that five phenolic chemicals known as nonenzymatic antioxidant agents alter dramatically in the incompatible and compatible systems that follows the gene for gene interaction. Ferulic acid was significantly increased in plants infected with IPO323 when compared to levels measured in plants inoculated with *ΔAvrStb6#33*. At 2-4 dpi, 42-41 ppm were found before falling to 25 ppm at 8 dpi (**Figure 8A**) and increasing back to 42 ppm between 12-21 dpi. Between 2 and 8 dpi, Apigenin fell during the incompatible interaction. The level of Apigenin remained stable during the remaining sample time-points (8-21 dpi) and was comparable between each of the investigated interactions (**Figure 8B**). The highest amount of Rutin (3.5 ppm) was found in the incompatible interaction at 4 dpi (**Figure 8C**). Rutin remained at stable levels over the other timepoints for both interactions. When compared to the incompatible interaction, both 2.5 dhb and coumaric acid followed the same pattern in that they are high in the compatible interactionat 4 dpi. 2.5 dhb and coumaric acid levels were 18.5 ppm and 4.3 ppm, respectively (**Figures 8D, E**). Finally, the activity of proline as non-enzymatic antioxidant agent was assessed in the studied interactions. Proline was elevated in the incompatible interaction between 2-8 dpi compared with its level in the compatible interaction (**Figure 8F**).

### AvrStb6-Stb6 maintains the integrity of plasma membrane

**Figure 9:**
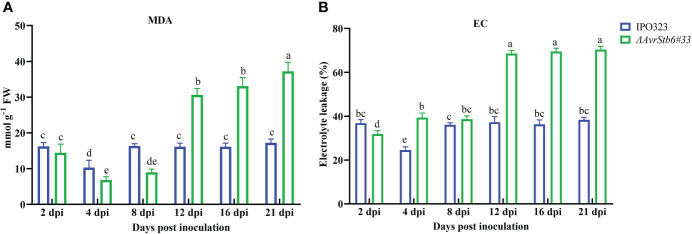
AvrStb6-Stb6 interaction impacts the **(A)** malondialdehyde (MDA) content, and **(B)** electrolyte leakage (EC) parameter measured in cv. Shafir infected by WT IPO323 or ΔavrStb6#33 strains. Data are indicate as mean ± SD (Standard Deviation) of four biological samples from one independent experiment. Different lowercase letters indicate statistically significant differences (P≤0.05).

The MDA content and EC parameter were used to explore the effects of the AvStb6/Stb6 on lipid peroxidation and membrane integrity. The MDA content in the compatible interaction increased steadily from 4 dpi, peaking at 21 dpi, but that of the incompatible interaction remained relatively unchanged over 2-21 dpi (except for a dip at 4 dpi) (**Figure 9A**). The EC index follows a similar trend as recorded for the MDA content of investigated interactions (**Figure 9B**).

## Discussion

Here, we compared some physiological and biochemical events that occurred in the cv. Shafir while responding differently to inoculation by IPO323 (carrying *AvrStb6*) or *ΔAvrStb6#33* (knock-out for *AvrStb6*) (**Supplementary Figure 1**). This system follows the gene-for-gene (GFG) model in which the indirect recognition of AvrStb6 *via* Stb6 results in an immune response (Kema et al., 2018; Saintenac et al., 2018).

The intriguing finding was that Stb6 temporarily mediates stomatal closure at an early point (2 dpi) presumably to prevent fungal penetration at the stomatal gate. This finding was confirmed by measuring the pore width and g_s_ of the treated leaves over time (**Figure 1**). This outcome was consistent with a recent study that found Stb16q mediates an early and temporary stomatal closure in response to an avirulent *Z. tritici* isolate (Battache et al., 2022). Typically, each stomatal pore is surrounded by two guard cells, that are extremely specialized in structure and function, and variations in the turgor pressure of these cells regulate the pore aperture size. Stomata are the potential gates for the entrance of pathogenic agents such as fungi and bacteria, and they are closed to prevent the pathogen’s entry into the plant tissues. It is worth mentioning that recent evidence has demonstrated that both salicylic acid (SA) and abscisic acid (ABA) pathways play central roles in stomatal closure (Zhang et al., 2008), which could be an intriguing subsequent project to investigate the impacts of these pathways in the examined interaction.

This study shows that when the AvrStb6-Stb6 interaction is lost, the ChlF transient curve of the *ΔAvrStb6#33*-infected leaves observed at 16 and 21 dpi rapidly and sharply declines (**Supplementary Figure 4**). These times correspond to the necrotrophic stage, during which the STB necrotic lesions enlarge, and the entire leaf turns brown and dies. F_O_, indicating changes in PSII antenna proteins (protein D1) (Schäfer et al., 1993) and its fluctuations reflecting modification of PSII photochemical reactions (Kalaji et al., 2017), declined sharply in the compatible interaction at 16 and 21 dpi (**Supplementary Table 1**). These events coincided with extending necrotic lesions covered by numerous pycnidia, which can be associated to extreme damage to the antenna proteins. Maximum quantum yield of photosystem II (F_V_/F_M_) is a reliable, indirect marker for studying stressed plants, with values of around 0.83 for healthy unstressed plants. A decrease in this parameter (lower than 0.8) indicates photoinhibition, oxidative burst, or PSII down-regulation (Murchie and Lawson, 2013). Our results showed that the value of F_V_/F_M_ decreased significantly from 0.79 to 0.70 in the incompatible interaction at 8 dpi. Afterward, this returned to the original level (0.79) without remarkable changes by 21 dpi. This finding suggested that during the incompatible interaction plants undergo a significant shock at the transition phase (8 dpi). Following on, plants could resist and maintain their PSII performance (**Figure 2A**). Additionally, the F_V_/F_M_ of plants during the compatible interaction completely stopped at 16 and 21 dpi due to irreversible damage occurring in the reaction centers, thereby leading to the complete annihilation of the photosynthetic apparatus. Our results were in agreement with the previous reports demonstrating a decrease in F_V_/F_M_ by 40% in the susceptible wheat cv. Enola challenged with *Z. tritici* (Mihailova et al., 2019). Other studies revealed that infection by *Colletotrichum gloeosporioides* and *C. lindemuthianum* led to the drop of F_V_/F_M_ below the equipment threshold, when massive tissue colonization and cell death occurred (Bassanezi et al., 2002; Otero-Blanca et al., 2021). Additionally, infection of susceptible wheat plants by *Bipolaris sorokiniana* led to a progressive reduction of F_V_/F_M_ matched up with the expansion of the lesions (Rios et al., 2017). Performance index (PI_abs_) could sensitively represent the functionality of both PSII and PSI providing quantitative data, exhibiting the current physiological state of plant performance under environmental stress (Strasser et al., 2004). We observed a similar trend of PI_abs_ fluctuations among the investigated treatment to that observed for F_V_/F_M_ (**Figure 2B**). This finding suggested that during the incompatible interaction resistant plants could inhibit *Z. tritici* infection at 8 dpi, hindering further STB symptom development. Contrastingly, this component declined dramatically in the compatible interaction at 21 dpi since all reaction centers of PSII were destroyed due to infection by *Z. tritici*. Previously, it was demonstrated that *(blank)*infection of wheat by *B. sorokiniana* led to the reduction of PI_abs_ by 28% at 14 dpi compared to reaction centre (RC) of non-inoculated plants (Matorin et al., 2018). The ABS/RC represents the average antenna size and expresses the total number of photons absorbed by PSII antenna chlorophylls divided by the total number of active RCs. The depression of some active RCs due to environmental stresses can lead to ABS/RC elevation while destroying the RCs can result in a decrease this parameter (Straaer, 1995). Our finding (**Figure 3A**) aligned with a report revealing that the ABS/RC index in wheat leaves infected by *B. sorokiniana* was elevated by 25% compared to the control at 14 dpi, once disease symptoms started to initiate  (Matorin et al., 2018). ET_0_/RC defines as electron transport flux in an active RC of PSII and reflects the functionality of an active RC (Force et al., 2003). The pattern of ET_0_/RC recorded in the incompatible interaction indicated that plants withstood the imposed infection by *Z. tritici* IPO323 at 8 dpi and recovered the functionality of their active RCs (**Figure 3B**). Dramatic reduction of ET_0_/RC at the necrotrophic stage of a compatible interaction in susceptible plants corroborated that there is no active RC in the PSII since the fungus kills all living cells to gain nourishment from the dead material (Kema et al., 1996). Our data were supported by the report that the ET_0_/RC declined significantly in the highly susceptible wheat cv. Tika Taka infected by *Fusarium graminearum* at 10 dpi (Katanić et al., 2021). DI_0_/RC indicates the dissipated energy flux mainly as heat per PSII reaction center, and this value is affected through the ratios of active/inactive RCs. An increase in this parameter demonstrated that the number of inactive RCs of PSII increased (Strasser et al., 2004). Our finding on changing the DI_0_/RC ratio (**Figure 3D**) verified that numerous active RCs become silenced as the *Z. tritici* fungus causes extensive necrotic lesions on the inoculated leaves. Furthermore, this parameter reached below the device threshold (0) during the compatible interaction, at 16 and 21 dpi, which coincides with the time the entire leaves became necrotic, and no active RCs exist in the PSII. These data were supported by the study showing that the Fusarium head blight (FHB) caused an increase in the DI_0_/RC in the winter wheat variety Tika Taka at 10 dpi (Katanić et al., 2021). NPQ is a central photoprotective tactic aiming to aid the plants’ ability to cope with the molecular damage caused by excess absorbed light energy. This event leads to the inactivation of the biochemical process that happens in the active RCs of PSII and down-regulates the photosynthetic activity (Ruban, 2016). Our analysis showed that the NPQ value was down-regulated remarkedly in plants infected by *ΔAvrStb6#33* at 16-21 dpi (**Figure 4**). This timing coincides with STB symptom expression as the emergence of the chlorotic and necrotic lesions on the inoculated leaves. This finding stated that NPQ plays a pivotal role as a photoprotective mechanism to prevent the induction of ROS accumulation as this parameter remained unchangeable in the resistant plants against *Z. tritici*. This finding agreed with the previous reports validating that *Z. tritici* infection led to a significant enhancement of NPQ value in the highly resistant wheat cv. Ariana at the necrotrophic phase (23 dpi). In contrast, the NPQ was remarkedly dampened in the highly STB susceptible wheat cv. Enola by 73% at 23 dpi (Mihailova et al., 2019). Our discovery followed a report that NPQ was significantly downregulated in soybean leaflets challenged with *C. truncatum* from 36 to 120 hai, which corresponds to an increase in anthracnose severity on the main vein (Dias et al., 2018).

Our finding demonstrated that the correlation between photosynthetic metrics of the leaves in the *ΔAvrStb6#33* (compatible interaction) was strongly positive, with the exception of ϕDo, which was negatively correlated with all photosynthesis parameters. However, in the IPO323-infected plants, the correlation of photosynthesis parameter and electron transfer chain (ETC) dropped drastically (**Supplementary Figure 5**).

Our histopathological study suggested that H_2_O_2_ is a pivotal substance to restrict and halt the fungal growth in the incompatible interaction involving AvrStb6-Stb6. As *Z. tritici* is a hemibiotroph, this fungus exploits two distinct pathogenicity stages, including biotrophic and necrotrophic phases, to complete its infection cycle (Kema et al., 1996). We observed that H_2_O_2_ accumulated in the plants infected by IPO323 at the biotrophic stage (**Figure 5A**). This observation corroborated its potential role in stopping the fungal growth similar to that documented previously for the biotrophic fungal pathogens such as *Blumeria graminis* f.sp. *hordei* (Thordal‐Christensen et al., 1997) and also *Z. tritici* (Shetty et al., 2003). Following the transition phase, massive H_2_O_2_ accumulation took place in the mesophyll cells during the compatible interaction coinciding with STB symptom expression and initiation of the tissue collapse. This event was observable in the plant inoculated by *ΔAvrStb6#33* by eye (**Figure 5A**). Therefore, *Z. tritici* may hijack the defence system at the necrotrophic growth stage similar to *Botrytis cinerea* to facilitate the infection process (Govrin and Levine, 2000).

Enzymatic antioxidants such as SOD, CAT, GPX, APX, and GR, are key players in maintaining the balance between the generation and degradation of ROS molecules. These chloroplast-produced enzymes are essential for plant antioxidant defence. Except for GPX, all of the investigated antioxidant enzymes were significantly stimulated in the incompatible interaction at 2 dpi, indicating that they may play a role in detoxifying free radicals in an attempt to protect living cells from the damaging effects of the oxidative burst (**Figure 7**). We additionally quantified the amount of five phenolic compounds as nonenzymatic antioxidants. For instance, Ferulic acid was highly induced in the incompatible interaction at all time points, suggesting an essential role in rendering the resistance reaction by neutralizing free radicals such as H_2_O_2_ at the biotrophic growth stage and preventing the oxidative burst as suggested formerly (Boz, 2015). A significant increase of Ferulic acid in the stem of chickpea beyond the infection area caused by *sclerotium rolfsii* led to such a conclusion that this substance may play a role in preventing the infection of chickpea by *S. rolfsii* (Sarma and Singh, 2003). Rutin was also activated significantly in the incompatible interaction at 4 dpi, suggesting a potential role for this compound in providing resistance in the Shafir-IPO323 context. Rutin has antifungal activity toward phytopathogenic agents and possesses a powerful antioxidant capacity (Báidez et al., 2007; Ismail et al., 2015). Apigenin levels were specifically enhanced in the incompatible interaction at the early biotrophic stage (2 and 4 dpi). This chemical probably increases notably at the biotrophic stage, when WT IPO323 faces more H_2_O_2_, to induce antioxidant enzymes. A previous report showed that Apigenin-treated rice seedlings had lower levels of lipid peroxidation and H_2_O_2_ concentration due to increased antioxidant enzyme activity (Mekawy et al., 2018). Coumaric acid was significantly increased in the compatible interaction at 4 dpi, followed by a sharp decline by 21 dpi. We concluded that this induction is a general response to infection, or this phenolic compound may be triggered by oxidative stress in the incompatible interaction to halt the fungal growth (Ansari et al., 2013). We noticed that during the compatible interaction the plant traded energy between defence and growth because this phenolic acid was low during the necrotrophic phase (**Figure 8**). However, there is accumulating evidence demonstrating that Coumaric acid plays a role in providing a resistance response in host-microbe interactions (Morales et al., 2017). We additionally discovered that the proline content, a known non-enzymatic antioxidant agent, was overproduced in the incompatible context, implying proline content may play a role in establishing the resistance response in the AvrStb6-Stb6 interaction (**Figure 8F**).

Our findings showed that the MDA content, a valuable biomarker for lipid peroxidation, and the EC parameter, a hallmark for cellular damages, gradually increased in the compatible interaction and peaked at 21 dpi, corresponding to massive tissue collapse and widespread cell death. These findings were consistent with those of a previous study comparing susceptible and resistant wheat genotypes inoculated with *Z. tritici* (Mihailova et al., 2019). This indicated that the AvrStb6-Stb6 interaction plays a pivotal role in maintaining the membrane integrity and preventing cellular electrolyte leakage

To sum up, a variety of methodologies were used to investigate the GFG model system where Stb6 recognizes AvrStb6 and causes an immune response in the *Z. tritici*-wheat pathosystem, concluding that several mechanisms are involved in mounting defence reactions. The resistant interaction between AvrStb6 and Stb6 is crucial for stomatal opening/closure and maintaining the efficiency of the photosynthetic apparatus. Preserving membrane integrity and regulating the level of oxidative stress are crucial for the resistance response. We recommend that future studies take advantage of this system providing two distinct phenotypes to discover defense-related genes or compounds using RNA-seq and metabolomics approaches.

## Data availability statement

The original contributions presented in the study are included in the article/**Supplementary Material**. Further inquiries can be directed to the corresponding author.

## Author contributions

AMG designed, supervised, and coordinated the study. FGN, MI, and EZ performed the infection assay. FGN conducted the infection biology, DAB and tryptophan assays and was involved in the photosynthetic related-assay. MI conducted the experiment related to HPLC and spectrophotometry. FD performed and analyzed the assay associated with photosynthetic part. AE conducted and analyzed the spectrophotometric assays. MAG employed PlantCV tool to analysis images associated with DAB and tryptophan blue staining. AG wrote the manuscript with substantial input from SA, MF, MJ-N, and AF. All authors contributed to the article and approved the submitted version.

## Funding

We acknowledge financial support from the Iran National Science Foundation Grant NO 97011958.

## Acknowledgments

We thank the University of Tehran for supporting the current study and providing the required facilities. We also would like to thank Prof. Gert Kema, University of Wageningen, for providing the IPO323 WT and the mutant lacking the AvrStb6.

## Conflict of interest

The authors declare that the research was conducted in the absence of any commercial or financial relationships that could be construed as a potential conflict of interest.

## Publisher’s note

All claims expressed in this article are solely those of the authors and do not necessarily represent those of their affiliated organizations, or those of the publisher, the editors and the reviewers. Any product that may be evaluated in this article, or claim that may be made by its manufacturer, is not guaranteed or endorsed by the publisher.

## Supplementary material

Supplementary Figure 1 AvrStb6-Stb6 interaction establishes immunity response. **(A)** Fully expanded first leaves were inoculated with the WT IPO323 or *ΔAvrStb6#33* strains by a hand sprayer** **(B)** The percentage of necrotic area formed on the inoculated leaves. (C) The percentage of pycnidia produced on the necrotic lesions. Pictures were taken at 2, 4, 8, 12, 16, and 21 days post-inoculation. Error bars are SD.

Supplementary Figure 2 Spider plot of the chlorophyll fluorescence parameters in wheat cv. Shafir inoculated by distilled water (Mock), WT IPO323, and *ΔAvrStb6#33* after 2, 4, 8, 12, 16, and 21 days post-inoculation.

Supplementary Figure 3 A schematic model illustrating changes that occurred in the photosynthetic parameter in cv. Shafir inoculated by IPO323 WT or *ΔAvrStb6#33* along with the Chlorophyll fluorescence images of infected wheat leaves taken at 2, 4, 8, 12, 16, and 21 dpi. Increases or declines in parameter’s amount are represented by size changes of boxes embedded each parameter.

Supplementary Figure 4 The impact of AvrStb6-Stb6 interaction on OJIP curve of cv. Shafir inoculated by distilled water (Mock), WT IPO323, and *ΔAvrStb6#33*. Data are shown as fluorescence intensity.

Supplementary Figure 5 Graphical representation of a correlation matrix of initial plant enzymatic and non-enzymatic antioxidant as well as photosynthesis parameters in cv. Shafir inoculated either with *ΔAvrStb6#33* **(A)** or IPO323 **(B)**. Red color represents positive correlation whereas blue represents negative correlation. Color intensity is proportional to the correlation, which depicted in the legend at the bottom.
