# Supplementary figures and images for: Stb6 mediates stomatal immunity, photosynthetic functionality, and the antioxidant system during the Zymoseptoria tritici-wheat interaction
Source: Front Plant Sci. 2022 Oct 26;13:1004691. doi: 10.3389/fpls.2022.1004691 (PMC9645118; doi:10.3389/fpls.2022.1004691)

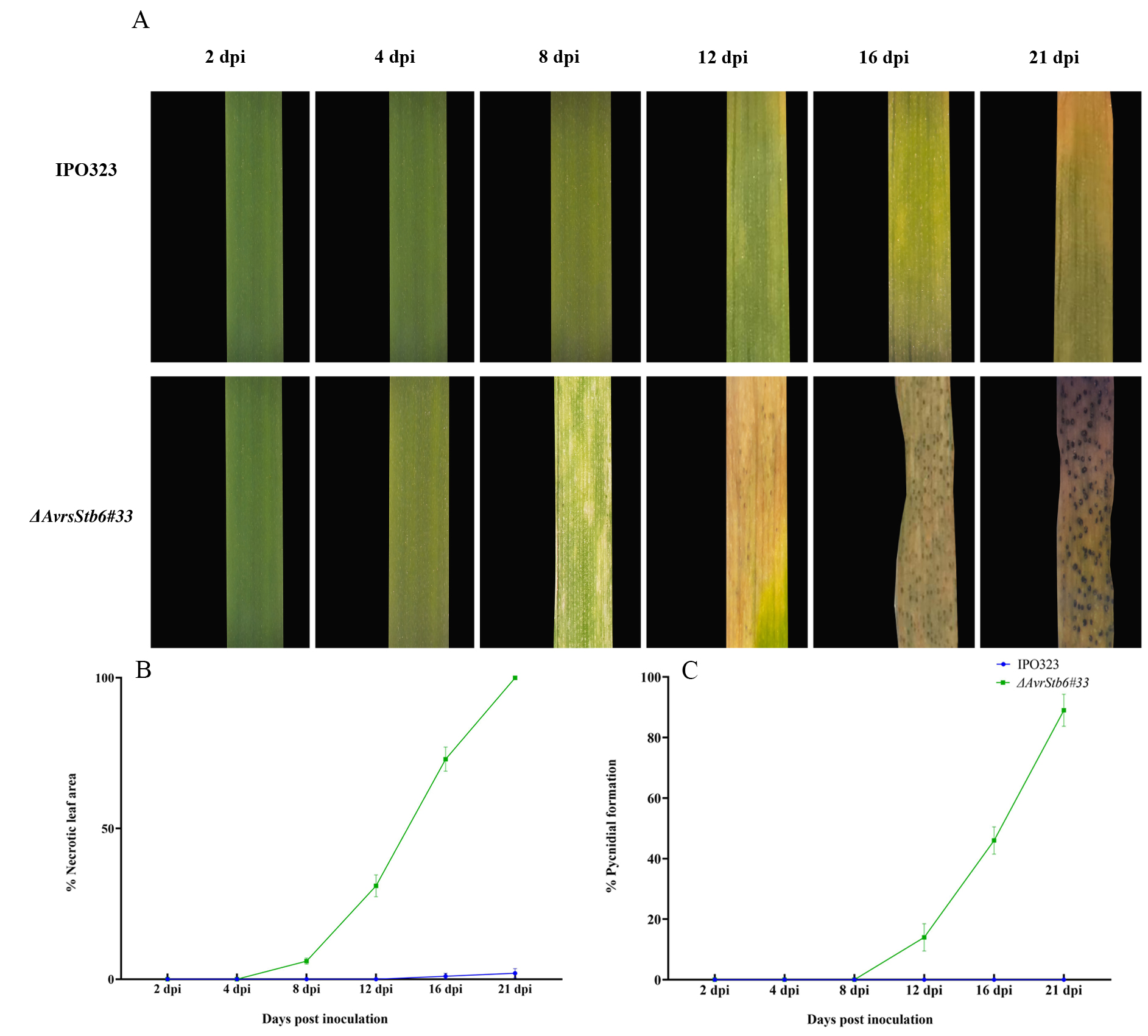

Supplement: Supplementary Figure 1 — AvrStb6-Stb6 interaction establishes immunity response. (A) Fully expanded first leaves were inoculated with the WT IPO323 or ΔAvrStb6#33 strains by a hand sprayer (B) The percentage of necrotic area formed on the inoculated leaves. (C) The percentage of pycnidia produced on the necrotic lesions. Pictures were taken at 2, 4, 8, 12, 16, and 21 days post-inoculation. Error bars are SD. [file Image_1.jpeg]

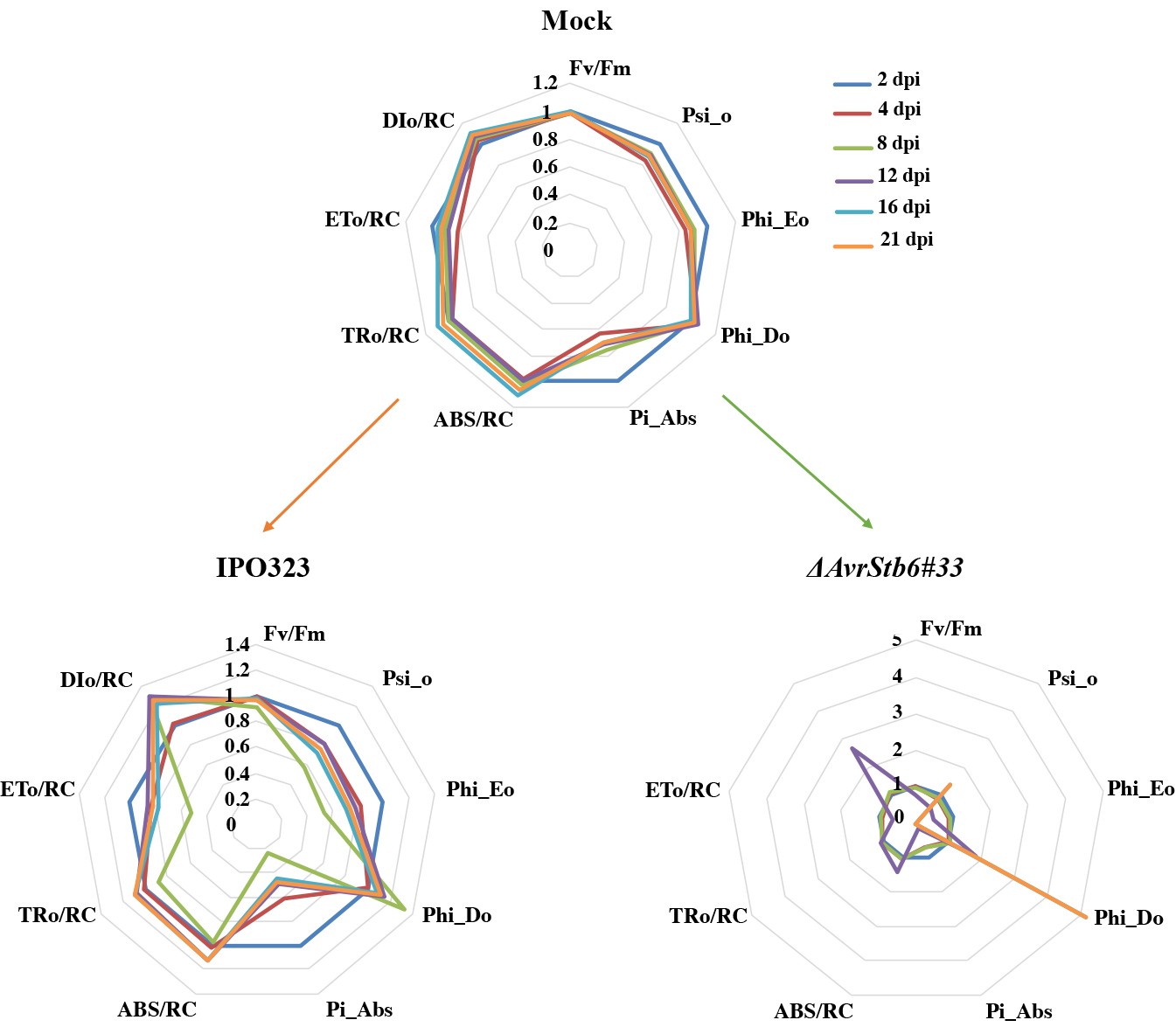

Supplement: Supplementary Figure 2 — Spider plot of the chlorophyll fluorescence parameters in wheat cv. Shafir inoculated by distilled water (Mock), WT IPO323, and ΔAvrStb6#33 after 2, 4, 8, 12, 16, and 21 days post-inoculation. [file Image_2.jpeg]

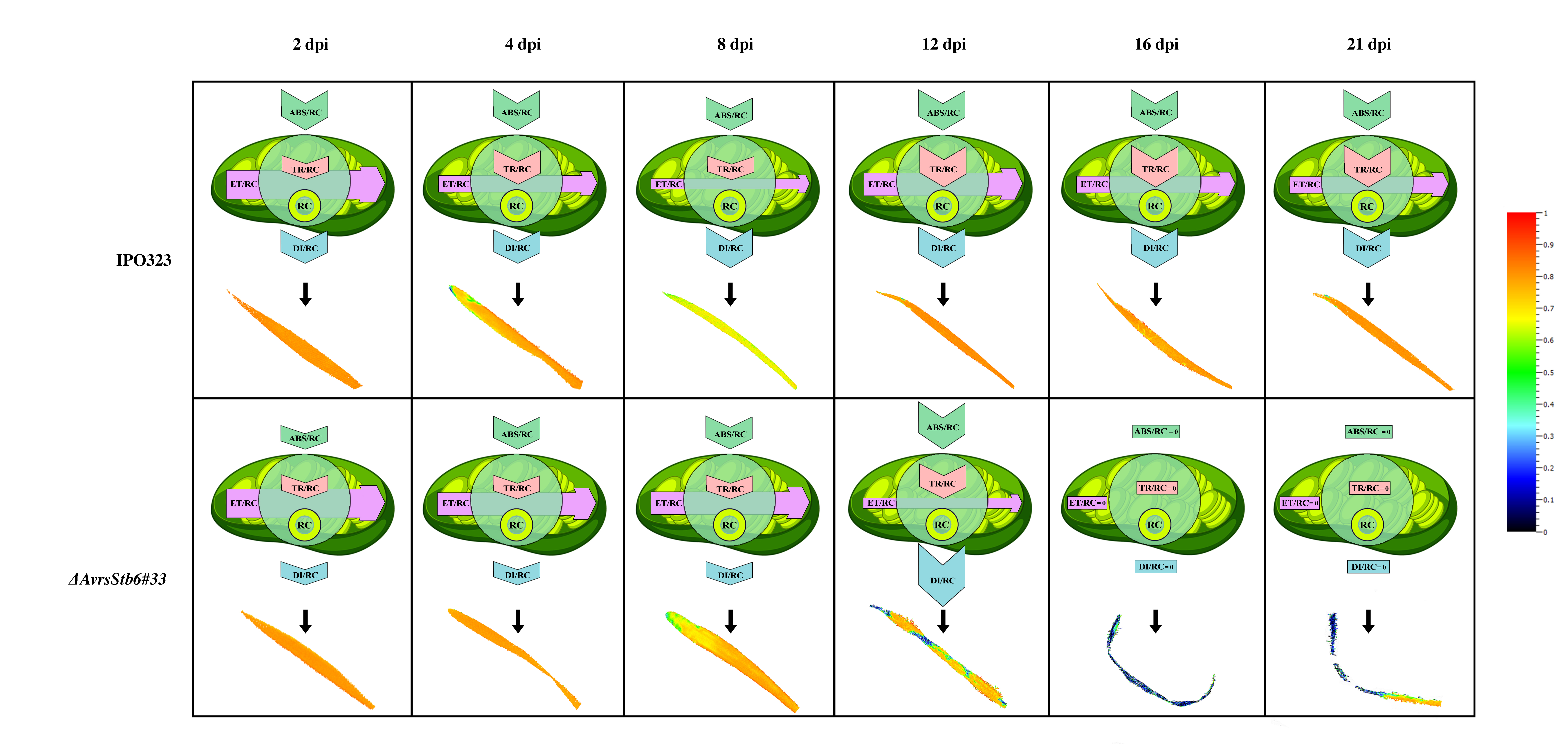

Supplement: Supplementary Figure 3 — A schematic model illustrating changes that occurred in the photosynthetic parameter in cv. Shafir inoculated by IPO323 WT or ΔAvrStb6#33 along with the Chlorophyll fluorescence images of infected wheat leaves taken at 2, 4, 8, 12, 16, and 21 dpi. Increases or declines in parameter’s amount are represented by size changes of boxes embedded each parameter. [file Image_3.jpeg]

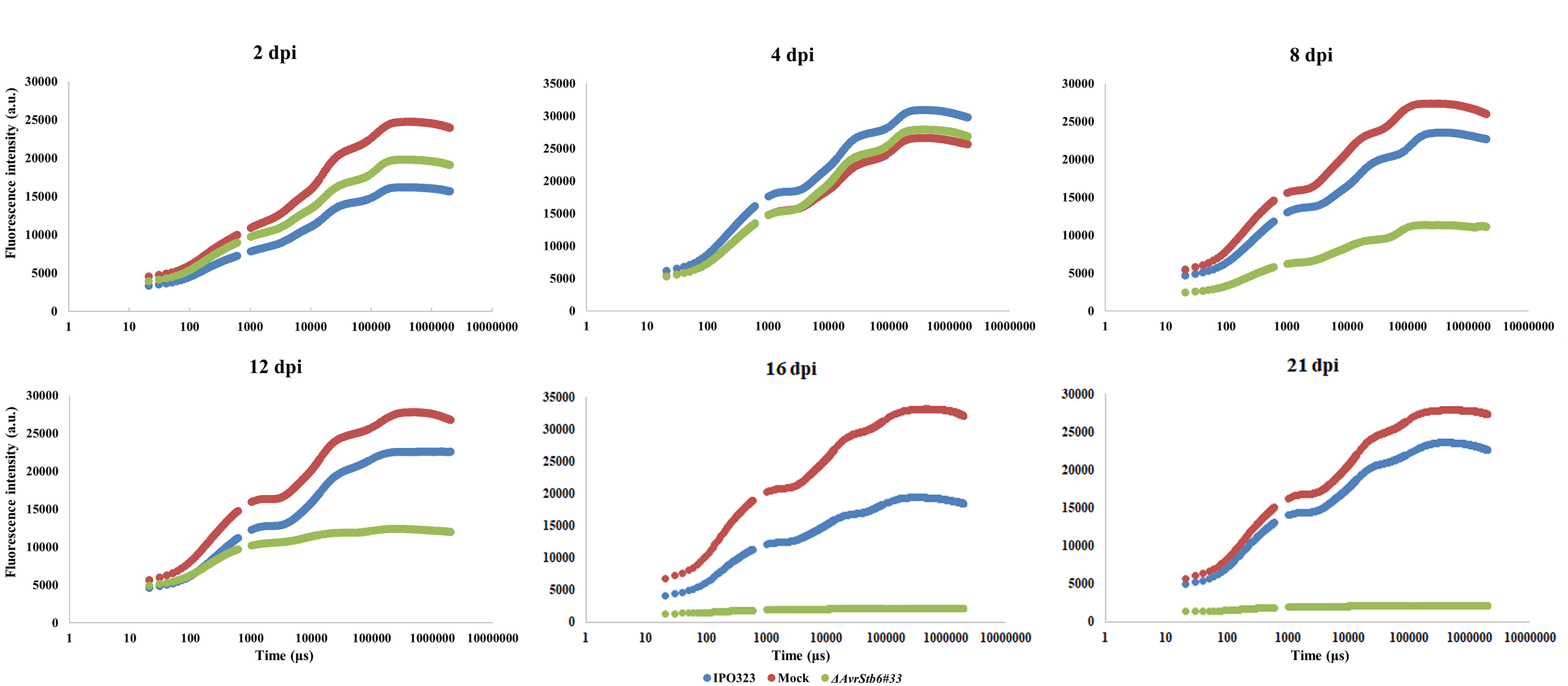

Supplement: Supplementary Figure 4 — The impact of AvrStb6-Stb6 interaction on OJIP curve of cv. Shafir inoculated by distilled water (Mock), WT IPO323, and ΔAvrStb6#33. Data are shown as fluorescence intensity. [file Image_4.tif]

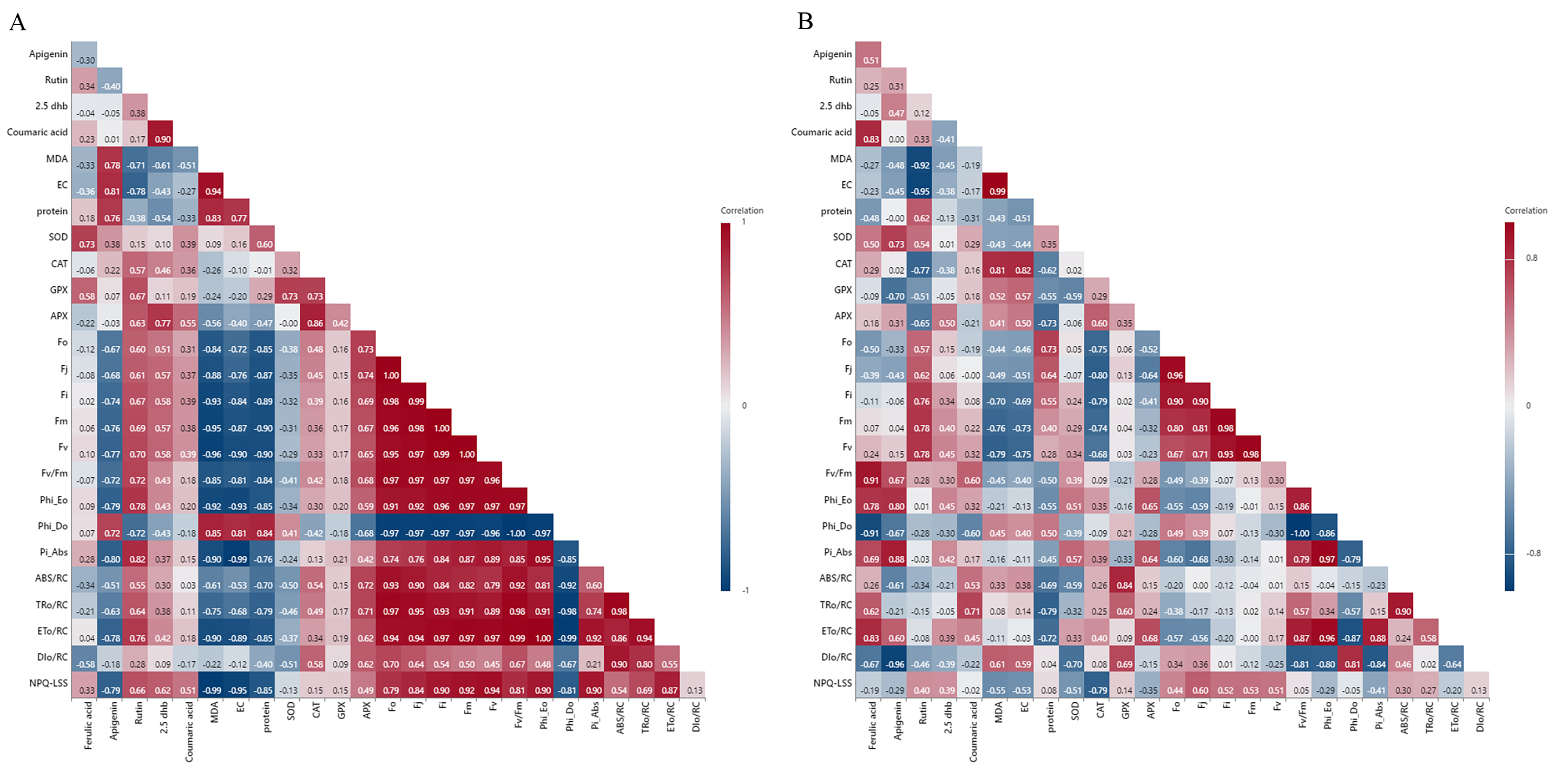

Supplement: Supplementary Figure 5 — Graphical representation of a correlation matrix of initial plant enzymatic and non-enzymatic antioxidant as well as photosynthesis parameters in cv. Shafir inoculated either with ΔAvrStb6#33 (A) or IPO323 (B). Red color represents positive correlation whereas blue represents negative correlation. Color intensity is proportional to the correlation, which depicted in the legend at the bottom. [file Image_5.jpeg]
