# Supplementary material for: Stb6 mediates stomatal immunity, photosynthetic functionality, and the antioxidant system during the Zymoseptoria tritici-wheat interaction
Source: Front Plant Sci. 2022 Oct 26;13:1004691. doi: 10.3389/fpls.2022.1004691 (PMC9645118; doi:10.3389/fpls.2022.1004691)
Supplement: Supplementary file 6 [file Table_1.docx]

| Abbreviation | Equation | Definition | | |
| --- | --- | --- | --- | --- |
| F_V_/F_o_ | ϕ P_0_/(1 − ϕ P_0_ ) | | Maximum efficiency of the water diffusion-reaction on the donor side of PSII |  |
| F_V_/F_M_ | TR_0_/ABS = [1 − (F_0_ /F_M_ )] | | Relative maximal variable fluorescence |  |
| Ψ_0_ | ET_0_ /TR_0_ = (1 − V_J_ ) | | The probability that a trapped exciton proceed an electron through ETC beyond Q_A_^-^ |  |
| ϕE_0_ | ET_0_ /ABS = [1 − (F_0_ /F_M_ )] ψ_0_ = φ P_0_· ψ_0_ | | Quantum yield of electron transport (at *t* = 0) |  |
| ϕD_0_ | 1 – ϕP_0_ = (F_0_/FM ) | | Quantum yield of energy dissipation |  |
| ɸ_PAV_ | ϕ P_0_ (1 − Vav) = ϕ P_0_ (Sm / tF_M_) | | The average quantum yield of primary photochemical reactions (from time 0 to F_M_). |  |
| ABS/RC | M_0_ (1/VJ)(1/ϕ P_0_) | | Light absorbance flux for PSII antenna Chlorophylls per reaction center |  |
| TR_0_/RC | M_0_ (1/V_J_) | | Trapped energy flux per reaction center |  |
| ET_0_/RC | M_0_ (1/V_J_) ψ_0_ | | Electron transport flux per reaction center |  |
| DI_0_/RC | (ABS/RC) − (TR_0_/RC) | | Energy flux not intercepted by an RC, dissipated in the form of heat, fluorescence, or transfer to other systems, at time t = 0. |  |
| abs | (RC/ABS) × (ϕP_0_/(1 –ϕP_0_)) × (ψ_0_/(1 – ψ_0_)) | | Performance index per absorbed light |  |

Supplementary Table1. Measured parameters related to the OJIP test.
