# Supplementary material for: Stb6 mediates stomatal immunity, photosynthetic functionality, and the antioxidant system during the Zymoseptoria tritici-wheat interaction
Source: Front Plant Sci. 2022 Oct 26;13:1004691. doi: 10.3389/fpls.2022.1004691 (PMC9645118; doi:10.3389/fpls.2022.1004691)
Supplement: Supplementary file 7 [file Table_2.docx]

| **No.** | **Phenolic compound** | **Migration time (minute)** | **UV λ max (nm)** |
| --- | --- | --- | --- |
| 1 | 2,5-dihydroxybenzoic acid | 17 | 327 |
| 2 | p-coumaric acid | 22.6 | 310 |
| 3 | Ferulic acid | 22.8 | 323 |
| 4 | Rutin | 27 | 257 |
| 5 | Apigenin | 38.6 | 267 |

Supplementary table 2. Features of five phenolic compounds traced in this study.
