# Supplementary material for: Stb6 mediates stomatal immunity, photosynthetic functionality, and the antioxidant system during the Zymoseptoria tritici-wheat interaction
Source: Front Plant Sci. 2022 Oct 26;13:1004691. doi: 10.3389/fpls.2022.1004691 (PMC9645118; doi:10.3389/fpls.2022.1004691)
Supplement: Supplementary file 8 [file Table_3.docx]

|  | **Days post inoculation** | **F_0_** | **F_i_** | **F_j_** | **F_v_** | **F_m_** | **ΦE_o_** | **ΦD_o_** |
| --- | --- | --- | --- | --- | --- | --- | --- | --- |
| Control | **2dpi** | 5200 | 21276 | 12532.5 | 20009.5 | 25209.5 | 0.5035 | 0.206 |
|  | **4dpi** | 5754.75 | 22301.5 | 15353.75 | 20746.25 | 26501 | 0.421 | 0.21675 |
|  | **8dpi** | 5999.625 | 24580 | 15643.94 | 22514.06 | 28513.69 | 0.453167 | 0.212271 |
|  | **12dpi** | 5663.75 | 23369.5 | 14803.5 | 21059.5 | 26723.25 | 0.4455 | 0.219 |
|  | **16dpi** | 7380 | 31373 | 19886 | 28241 | 35621 | 0.442667 | 0.207333 |
|  | **21dpi** | 6697.875 | 28316.75 | 17871.38 | 25453.5 | 32151.38 | 0.444917 | 0.212208 |
| IPO323 | **2dpi** | 3672.667 | 14234 | 8534.667 | 13249.67 | 16922.33 | 0.496 | 0.222 |
|  | **4dpi** | 5779 | 23010.25 | 15655.25 | 20794.75 | 26573.75 | 0.4075 | 0.22075 |
|  | **8dpi** | 5793 | 18592.75 | 14617.75 | 13848 | 19641 | 0.2625 | 0.2955 |
|  | **12dpi** | 5470.917 | 20274.83 | 14251 | 17797.83 | 23268.75 | 0.38525 | 0.25325 |
|  | **16dpi** | 4045 | 14636.25 | 11480.25 | 12871.25 | 16916.25 | 0.35 | 0.239 |
|  | **21dpi** | 5200 | 17455.54 | 12865.63 | 15334.54 | 20092.5 | 0.367625 | 0.246125 |
| *ΔAvrStb6#33* | **2dpi** | 4320.667 | 17610.67 | 10495.33 | 17407.33 | 21728 | 0.513667 | 0.201333 |
|  | **4dpi** | 5690.75 | 23697.75 | 15261.75 | 22082 | 27772.75 | 0.45175 | 0.20425 |
|  | **8dpi** | 5477.375 | 21788.75 | 13943.13 | 20377.88 | 25855.25 | 0.46225 | 0.211375 |
|  | **12dpi** | 4743.917 | 13644.21 | 10771.92 | 9954.292 | 14698.21 | 0.239708 | 0.365875 |
|  | **16dpi** | 1162.5 | 1127.25 | 1157.5 | 0 | 1117.75 | 0 | 1.0385 |
|  | **21dpi** | 1080.125 | 1047.688 | 1075.625 | 0 | 1039.063 | 0 | 1.038125 |
| ***P* value** | **Treatment** | <.0001^**^ | <.0001^**^ | <.0001^**^ | <.0001^**^ | <.0001^**^ | <.0001^**^ | <.0001^**^ |
|  | **Days post inoculation** | 0.0145^*^ | 0.0134^*^ | 0.0136^*^ | 0.0054^**^ | 0.0078^**^ | <.0001^**^ | <.0001^**^ |
|  | **Treatment × Days post inoculation** | 0.0008^**^ | <.0001^**^ | 0.0004^**^ | <.0001^**^ | <.0001^**^ | <.0001^**^ | <.0001^**^ |

Supplementary Table 3. The table shows the effect of deleting *AvrStb6* on OJIP fluorescence kinetics (F_O_, F_i_, F_j_, F_v_ and F_m_), the quantum electron transfer function (Ф_E0_) and quantum energy dissipation function (Ф_D0_). The leaves of cv. Shafir harboring the Stb6 were inoculated with the IPO323 strain carrying the *AvrStb6* or *AvrStb6#33* without the *AvrStb6*. The OJIP transients of dark-adapted (20 min) non-inoculated and inoculated plants were measured at various time intervals, including 2, 4, 8, 12, 16, and 21 days post-inoculation.
