# Supplementary material for: Stb6 mediates stomatal immunity, photosynthetic functionality, and the antioxidant system during the Zymoseptoria tritici-wheat interaction
Source: Front Plant Sci. 2022 Oct 26;13:1004691. doi: 10.3389/fpls.2022.1004691 (PMC9645118; doi:10.3389/fpls.2022.1004691)
Supplement: Supplementary file 9 [file Table_4.docx]

|  | **Day 1** | | |  | **Day 2** | | |  |  | **Day 4** |  |  | **Day 8** | | |  | **Day 12** | | |
| --- | --- | --- | --- | --- | --- | --- | --- | --- | --- | --- | --- | --- | --- | --- | --- | --- | --- | --- | --- |
|  | IPO323 | *ΔAvrStb6#33* | Odd Ratio |  | IPO323 | *ΔAvrStb6#33* | Odd Ratio |  | IPO323 | *ΔAvrStb6#33* | Odd Ratio |  | IPO323 | *ΔAvrStb6#33* | Odd Ratio |  | IPO323 | *ΔAvrStb6#33* | Odd Ratio |
| Pct germinated Spores | 28.44 | 27.92 | 1.02^NS^ |  | 24.37 | 23.92 | 1.02 ^NS^ |  | 21.62 | 20.51 | 1.06^NS^ |  | 15.92 | 14.59 | 1.10^NS^ |  | 20.81 | 17.25 | 1.26 ^NS^ |
| Pct spores with germ tubes growing towards a stoma | 2.74 | 3.41 | 0.79 ^NS^ |  | 4.51 | 4.66 | 0.96 ^NS^ |  | 5.41 | 5.77 | 0.93 ^NS^ |  | 7.33 | 7.62 | 0.95 ^NS^ |  | 5.33 | 6.22 | 0.84 ^NS^ |
| Pct spores with germ tubes ending on a stoma | 1.26 | 1.41 | 0.89 ^NS^ |  | 2.96 | 3.03 | 0.97 ^NS^ |  | 4.51 | 4.59 | 0.98 ^NS^ |  | 6.51 | 6.51 | 1 ^NS^ |  | 4.81 | 5.03 | 0.95 ^NS^ |
| Pct spores with germ tubes growing past a stoma | 0.66 | 0.59 | 1.12 ^NS^ |  | 1.48 | 1.703 | 0.86 ^NS^ |  | 1.77 | 2.37 | 0.74 ^NS^ |  | 3.55 | 4.59 | 0.76 ^NS^ |  | 2.37 | 4.81 | 0.47 ^*^ |

Supplementary Table 4. Observed incidence of various developmental phases in the infection course of *Zymoseptoria tritici* IPO323 and *ΔAvrStb6#33* in leaves of the wheat cultivar Shafir. Values given are percentages.
